# Effects of *Qingke* β-glucan with different molecular weights on pasting, gelation, and digestive properties of rice starch

**DOI:** 10.1016/j.fochx.2023.100803

**Published:** 2023-07-21

**Authors:** Lan Zhao, Xinyan Jin, Jia Wu, Huibin Chen

**Affiliations:** a School of Food Science and Engineering, College of Life Sciences, Fujian Normal University, Fuzhou, China; b College of Biological Science and Engineering, Fuzhou University, Fuzhou, Fujian 350116, China

**Keywords:** *Qingke* β-glucan, Molecular weights, Confocal laser scanning microscopy, Pasting properties, In *vitro* starch digestibility

## Abstract

This study aimed to investigate the impact of *Qingke* β-glucan (QBG) concentrations and molecular weights (MWs) on rice starch (RS). With the increasing concentrations and MWs, the pasting properties and gelatinization enthalpy of RS/QBG suspension decreasing was observed by using rheometer and differential thermal scanning analysis, respectively, which was consistent with the results of X-ray diffraction. In Infrared spectrum, QBG combined with leached amylose via hydrogen bonds, thus preventing the reaggregation of RS particles and inhibiting the short-term retrogradation of RS. The results of scanning electron microscopy and confocal laser scanning microscopy suggested that interaction between QBG and RS changed RS microstructure, reduced the leached amylose of the starch, and thus altered RS/QBG digestibility that the digestion rate of RS/QBG decreased with the incrementing QBG MWs at in *vitro* simulated experiments. These results provide further understanding and expand potential application to starch-based foods.

## Highlights

• QBG concentrations and MWs impact on the RS structure and digestive properties.
• Pasting, gelation, and digestive properties were characterized.
• QBG addition decreased RS digestion rate and viscosity.
• The properties of QBG on the RS gels related with QBG viscosity.

## Introduction

Starch is a vital carbohydrate polymer in the food industry and has numerous applications, including health-promoting foods, food additives, cosmetics, and pharmaceuticals (Przetaczek-Rożnowska, 2017). Rice starch (RS) is white in color, bland in flavor, smooth, gel-like consistency, and hypoallergenic in many people (Bao & Bergman, 2018). The drawbacks of RS are similar to other cereal starches. It includes a tendency to retrograde gels and form unfavorable weak-bodied cohesive gels when exposed to high shear, acidic, or boiling temperatures. To address these drawbacks, researchers have evaluated the combination of native RS with other hydrocolloid polysaccharides (Banchathanakij and Suphantharika, 2009, Chen et al., 2017, Kim et al., 2006, Zheng et al., 2021).

Non-starch hydrocolloids are extensively used in starch-containing systems to improve the quality and stability of the final products because they can alter gelation and flow behavior (Cui et al., 2023). These hydrocolloids are extracted from natural sources. Hydrocolloids can be added to starches to increase their quality without chemical modifications. Most studies have focused on the effects of hydrocolloids on RS, such as β-glucans (Banchathanakij & Suphantharika, 2009) and pullulan (Chen et al., 2017), and the results have demonstrated that the addition of hydrocolloids might alter the rheology and gelatinization of RS.

β-glucan, as a natural polysaccharide hydrocolloid, has garnered significant interest from both the pharmaceutical and functional food industries due to its numerous benefits for human and animal health, besides its thickening or gelling capabilities. Hull-less barley (*Hordeum vulgare* L. *var. nudum* Hook.), also called *Qingke*, is a kind of cultivated barley with naked grains. *Qingke* is the main staple food crop in Qinghai-Tibet Plateau. Nowadays, it is gaining popularity as “functional grain” because it contains various biologically active ingredients, including 4–11% *Qingke* β-glucan (QBG) (Du et al., 2014). Our previous study showed that β-glucan is a homopolysaccharide composed of d-glucopyranose units connected by (1 → 3) and (1 → 4) glycosidic linkages (Zhao et al., 2022). QBG has rich functional properties and can potentially prevent and treat type 2 diabetes (Li et al., 2020), colon cancer, low cholesterol levels (Whitehead et al., 2014), and metabolic disorders (Cloetens et al., 2012). Moreover, β-glucan can form a gel and increase viscosity (Ahmad et al., 2012), enhancing the technological capabilities of the product. Increased gut viscosity decreases starch amylolysis and glucose absorption by reducing pancreatic amylase activity and transporting free sugars toward the gut wall.

The addition of β-glucan impacting on the physicochemical and digesting properties has been observed in previous studies. For example, Brennan and Cleary (2007) adding 5% β-glucan to bread could significantly lower the release rates of reducing sugar and the degradation of α-amylase and pepsin in *vitro* digestion. The influence of β-glucan on the viscosity of oat flour by the content, molecular weight, and structural characteristics of β-glucan has been investigated (Liu, 2010). Cui et al. (2023) reported that β-glucan with different molecular weights could obtain pea starch gels with diverse textures for multiple applications.

Few studies have been conducted on the effects of different molecular weights and concentrations of *Qingke* β-glucan on the physicochemical features of starch. Considering that rice and rice starch products are widespread in eastern countries, this study used RS as a model to investigate the interaction between starch and *Qingke* β-glucan. The effects of different concentrations and molecular weights of *Qingke* β-glucan on rice starch's rheological, pasting, and digestive properties were investigated. Moreover, we analyzed alterations in the gel microstructure between RS and QBG using scanning electron microscopy and confocal laser scanning microscopy. This study provides valuable insights into the potential applications of polysaccharides in improving starch-based food-processing capabilities and shelf life.

## Materials and methods

### Materials

RS containing 18.20% amylose, 0.10% lipids, 0.89% proteins, and 0.12% ash were purchased from Golden Agriculture Biotech. Co., Ltd. (Wuxi, China). A double-enzymatic technique was used to extract β-glucan from *Qingke* flour (Lazaridou et al., 2004), and acid hydrolysis was performed to obtain QBG with low molecular weight (Zhao et al., 2022). The QBG content was over 85.0% in all samples. *Qingke* β-glucans were composed of β-(1 → 3)-linked-d-glucopyranosyl, β-(1 → 4)-linked-d-glucopyranosyl. The high (HQBG), medium (MQBG), and low molecular weight (LQBG) β-glucans were 280 (*±*18), 190 (*±*11), and 155 (*±*12) KDa, respectively, and the macromolecular characteristics were characterized in our previous study (Zhao et al., 2022). Glucose Oxidase/Peroxidase Assay Kit (GOPOD) was purchased from Sigma-Aldrich. All other reagents and chemicals used in this study were of analytical grade.

### RS and RS/QBG system preparation

QBG solutions with different molecular weights (HQBG, MQBG, and LQBG) and concentrations (2.5% RS and 5.0% RS, w/w) were prepared. Then RS (5.0%, w/w) was added to the QBG solutions, the mixtures were swirled to create homogenous slurries. Then, the samples were split into groups named RS/5.0%HQBG, RS/5.0%MQBG, RS/5.0%LQBG, RS/2.5%HQBG, RS/2.5%MQBG, and RS/2.5 %LQBG.

### Determination of pasting properties

The pasting characteristics of the RS/QBG mixtures were determined using a Modular Compact 302 rheometer (Anton Paar, Austria) equipped with an intelligent starch cell. The RS concentration was 5.0%, and the QBG was 2.5% and 5.0% of the RS dry weight, respectively. RS powders and RS/QBG mixtures in the starch cells were dispersed in 20 mL of distilled water. For measurement, the slurries were incubated at 28 °C for 2 min and then gradually heated from 28 °C to 95 °C at a heating rate of 5 °C/min. The samples were maintained at 95 °C for 30 min, cooled from 95 °C to 50 °C at a cooling rate of 5 °C/min, and held at 50 °C for 10 min. The stirring speed was set at 800 rpm for the initial 10 s, followed by 160 rpm to confirm the diffusion consistency. Pasting parameters, including peak viscosity (PV), trough viscosity (TV), final viscosity (FV), breakdown viscosity (BD), setback viscosity (SB), were recorded using pasting curves.

### Rheological measurements

#### RS and RS/QBG paste preparation

The prepared RS and RS/QBG samples according to Section “RS and RS/QBG system preparation” were heated in a water bath at 95 °C for 20 min and then the RS and RS/QBG paste was cooled to room temperature for further analysis.

#### Dynamic rheological measurements

Dynamic oscillatory measurements were performed using a previously described protocol with slight modifications (Kong et al., 2020). An MCR 302 (Anton Paar, Austria) equipped with a 50 mm cone plate (CP-50) was used to investigate the rheological properties of the RS and RS/QBG paste. The samples were subjected to 0.5% strain at 25 °C and a frequency range of 0.1–100 rad/s. In addition, the samples' loss modulus (G'') and storage modulus (G') were measured.

### Differential scanning calorimetry

The gelatinization properties of RS were analyzed by differential scanning calorimetry (DSC) (Netzsch, DSC214, Germany). A 30.0% w/w (dry basis) mixture of RS and RS/QBG was selected by maintaining the QBG ratio at 2.5% and 5.0% RS. The thoroughly mixed solutions were initially hydrated for 24 h at room temperature. Subsequently, 10-15 mg of the solution was precisely weighed and loaded into an aluminum DSC pan, followed by immediate hermetical sealing to prevent moisture loss. An empty pan was used as a reference, and the sealed pan was heated from 20 to 100 °C at 10 °C/min. The onset temperature (*T*_o_), peak temperature (*T_p_*), end temperature (*T*_c_), and enthalpy (*ΔH*) of gelatinization were determined using the software.

### X-ray diffraction measurement

The RS and RS/QBG mixtures used in this experiment were the same as those described in Section “RS and RS/QBG paste preparation”. The mixtures were stored at −80 °C overnight and then transferred to a vacuum freeze-dryer for freeze-drying. After freeze-drying, the dried samples were milled and passed through a 100-mesh sieve. To ascertain the crystalline structure of the starch, we used an X-ray diffractometer (XRD) (Rigaku Ultima IV X, Japan) fitted with Cu-K radiation (λ = 0.15406 nm). The samples were scanned within the 4° < 2θ < 50° range using a scanning speed of 5°/min and 10 s for each step, with a tube pressure and flow of 40 kV and 40 mA, respectively.

### Fourier transform infrared spectroscopy

The samples used in this experiment were the same as those described in Section “RS and RS/QBG paste preparation”. After overnight storage at −80 °C, the samples were freeze-dried in a vacuum freeze-drier and passed through a 100-mesh sieve. The ratio of samples to KBr was approximately 1:120, and the samples were compressed into slices. A Nicolet IS50 Fourier transform infrared (FT-IR) spectrometer (Thermo Scientific, USA) was used to acquire FT-IR spectra. The test wavelengths ranged from 400 cm^−1^ to 4000 cm^−1^.

### Scanning electron microscopy

The RS (5%, w/w, dry basis) and RS/QBG (5.0% RS w/w, dry basis) mixtures used in this experiment were the same as those described in Section “RS and RS/QBG paste preparation”. The samples were stored at −80 °C overnight and freeze-dried in a vacuum freeze-drier. The granular starch morphology was observed using a Nova Nano SEM 230 field scanning electron microscope (SEM) (Czechia). The samples were gold-coated and fixed on conductive tape, operating at an accelerating voltage of 20 kV.

### Confocal laser scanning microscopy

The gel images were captured using a confocal laser scanning microscope (CLSM) (Lecia, Germany). A previously described procedure with minor modifications was used (Qiu et al., 2016). First, fluorescein 5-isothiocyanate (FITC) was dissolved in 100 mL of distilled water to obtain a FITC stock solution. Next, 100 µL of starch suspension, with or without QBG, was stained by combining it with 20 µL of FITC stock solution. Finally, the stained samples were placed on a glass slide and observed within 15 min. A He/Ne and Ar laser with an excitation wavelength of 488 nm was used for FITC, with an emission wavelength of 525 nm. In all experiments, the objective lens provided 20X magnification.

### *In vitro* digestion characteristics

With slight modifications, *in vitro* starch digestibility was measured using the Englyst method (Englyst & Cummings, 1985). Gelatinized starch granules (1.0 g, dry basis) with or without QBG were mixed with 20 mL of sodium acetate buffer (0.1 mol/L, pH 5.2), and 5 mL of enzyme working solution was added for digestion (the enzyme working solution was freshly prepared according to a previous study) (Qiu et al., 2016). Subsequently, digestion was performed for 20 and 120 min. The hydrolysate (0.5 mL) was mixed with 20 mL ethanol solution (70%, v/v) to denature the enzymes. The solution was centrifuged at 4500 rpm for 10 min by a H1850R centrifugation apparatus (Cence, China), and the glucose content in the supernatant was measured using a GOPOD kit. The following formulae were used to determine the amounts of rapidly digested starch (RDS), slowly digestible starch (SDS), and resistant starch (RS) in the samples:

$$RDS\left(\%\right)=\left(G_{20}-GF\right)\times 0.9/TS\times 100\%$$

$$SDS\left(\%\right)=\left(G_{120}-G_{20}\right)\times 0.9/TS\times 100\%$$

$$RS\left(\%\right)=\left[TS-\left(RDS+SDS\right)\right]\times 0.9/TS\times 100\%$$

G20 and G120 refer to the quantities of glucose generated 20 and 120 min after hydrolysis, respectively. On the other hand, GF represents the amount of free glucose present in the samples, whereas TS refers to the total dry weight of the samples.

### Statistical analysis

All tests were conducted at least in triplicate and the data was analyzed using IBM SPSS statistics version 22.0 (IBM, Armonk, NY, USA). Analysis of variance (ANOVA) was followed by the Turkey’s HSD test for pairwise multiple comparisons between treatments, and the significance level was set as *P* < 0.05. Images were constructed using the Origin 2021 software.

## Results and discussion

### Pasting properties

**Fig. 1:**
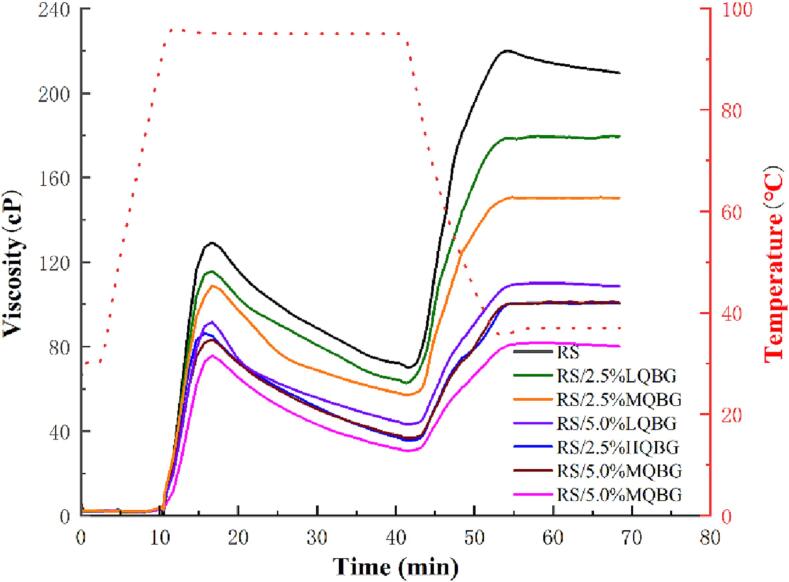
Pasting curves of RS and RS/QBG with β-glucan of different molecular weights and concentrations. RS, rice starch; LQBG, low *Qingke* β-glucan; MQBG, medium *Qingke* β-glucan; HQBG, high *Qingke* β-glucan.

The pasting curves and properties of RS and RS/QBG with different molecular weights and concentrations are shown in Fig. 1 and Table 1, respectively. According to the results, the addition of β-glucan decreased the starch paste's PV, TV, FV, BD, and SB. The peak viscosity is related to the amount of leached amylose, starch particle expansion, friction between the expanded particles, and competition for free water between the leached amylose and ungelatinized particles. After adding 5.0% LQBG, 5.0% MQBG, and 5.0% HQBG, the PV of RS/QBG was in the order of 91.5 cP > 84.7 cP > 75.6 cP. After adding 2.5% LQBG, 2.5% MQBG, and 2.5% HQBG, the PV of RS/QBG was in the order of 115.1 cP >108.8 cP > 86.3 cP. The FV of RS/QBG gradually decreased with an increase in concentration and molecular weight of QBG. The decrease in viscosity was due to insufficient starch granular swelling when QBG was wrapped around the RS during pasting, despite the high viscosity of β-glucan. Moreover, β-glucan, a linear molecule, inhibits the aggregation of amylose and amylopectin molecules during gelatinization, thereby reducing RS/QBG viscosity. This finding is consistent with the findings of two previous studies (Brennan and Cleary, 2007, Symons and Brennan, 2004) but contradicts the results of another study (Banchathanakij & Suphantharika, 2009).

**Table 1:** Pasting parameters of RS and RS/QBG systems with β-glucan of different molecular weights and concentrations.

Sample	PV (cP)	TV (cP)	FV (cP)	BD (cP)	SB (cP)
RS	129.6 ± 5.5*^a^*	70.2 ± 3.4*^a^*	216.9 ± 4.2*^a^*	59.5 ± 2.8*^a^*	146.8 ± 4.1*^a^*
RS/2.5 %LQBG	115.1 ± 4.4*^b^*	62.8 ± 2.6*^b^*	178.9 ± 3.4*^b^*	52.3 ± 2.2*^b^*	116.1 ± 4.4*^b^*
RS/2.5 %MQBG	108.8 ± 5.1*^b^*	57.1 ± 2.3*^c^*	150.5 ± 5.4*^c^*	51.7 ± 2.2*^b^*	93.4 ± 4.4*^c^*
RS/2.5 %HQBG	86.3 ± 4.1*^c^*	35.6 ± 1.6*^e^*	100.2 ± 3.6*^e^*	50.6 ± 2.3*^b^*	64.6 ± 2.8*^d^*
RS/5.0 %LQBG	91.5 ± 3.9*^c^*	43.2 ± 1.8*^d^*	109.7 ± 3.8*^d^*	48.3 ± 1.8*^bc^*	66.6 ± 2.2*^d^*
RS/5.0 %MQBG	84.7 ± 2.1*^c^*	36.9 ± 1.7*^e^*	100.2 ± 4.1*^e^*	47.9 ± 2.3*^bc^*	63.4 ± 2.1*^d^*
RS/5.0 %HQBG	75.6 ± 3.3*^d^*	30.7 ± 1.2*^f^*	80.3 ± 3.2*^f^*	44.9 ± 2.1*^c^*	49.6 ± 1.7*^e^*

RS, rice starch; LQBG, low *Qingke* β-glucan; MQBG, medium *Qingke* β-glucan; HQBG, high *Qingke* β-glucan; PV, peak viscosity; TV, through viscosity; FV, final viscosity; BD, breakdown viscosity; SB, setback viscosity. Results are presented as the mean ± SD. Values with different lowercase letters in the same column represent significant differences (p < 0.05).

BD represents the variation between the PV and TV in the pasting curve, which is indicative of the degree of damage during gelatinization and the thermal stability of starch paste (Zheng et al., 2021). After the addition of QBG, the BD of rice starch decreased from 59.5 to 44.9 cP, indicating that QBG inhibited the swelling ability of rice starch. This finding was related to the inhibition of rice starch gelatinization by QBG, which was verified by the decrease in PV. These results indicated that β-glucan significantly prevented starch damage at high concentrations and molecular weights. With a gradual increase in the concentration and molecular weight of β-glucan, part of the β-glucan molecule is adsorbed more tightly on the RS surface through hydrogen bonds. The other part was entangled with the permeated soluble starch molecules and gradually wrapped around the starch particle surface.

SB is the difference between FV and TV in the gelatinization curve, and the starch paste's viscosity increases during the cooling process. As shown in Table 1, the viscosity of the RS paste increased during cooling, suggesting RS is susceptible to temporary regeneration. The decrease in SB suggests that β-glucan may be linked with starch molecules, reducing the connection between amylose molecules. In other words, linear β-glucan molecules may prevent amylose molecules from rearranging in the starch paste, inhibiting the short-term regeneration of amylose. Consequently, there was a substantial decrease in the leaked soluble starch, specifically amylose, resulting in a significant decrease during the cooling phase, as verified by the decrease in PV. A previous study demonstrated that hydrocolloids inhibit retrogradation (Zheng et al., 2021).

### Rheological analysis

**Fig. 2:**
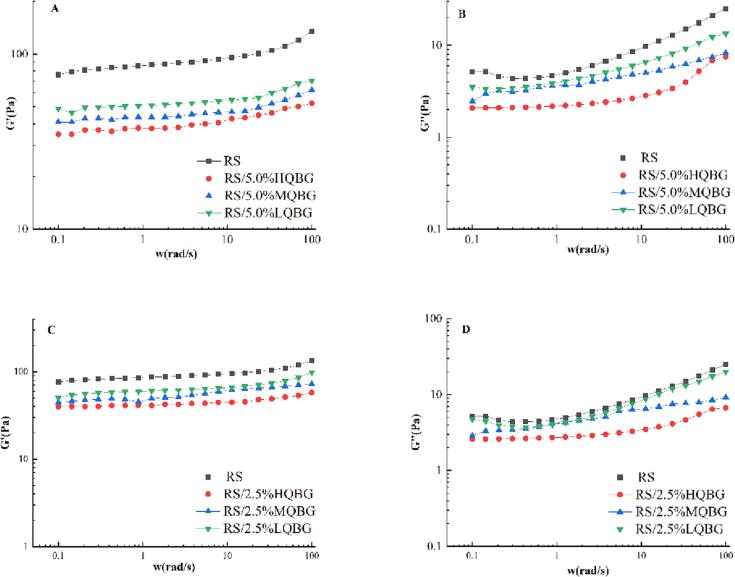
The rheological parameters of RS and RS/QBG with β-glucan of different molecular weights and concentrations. (A) G' of RS and RS/5.0%QBG, (B) G'' of RS and RS/5.0%QBG, (C) G' of RS and RS/2.5%QBG, (D) G'' of RS and RS/2.5%QBG. RS, rice starch; LQBG, low *Qingke* β-glucan; MQBG, medium *Qingke* β-glucan; HQBG, high *Qingke* β-glucan.

The effects of QBG on the storage modulus (G') and loss modulus (G'') of RS gel are illustrated in Fig. 2. All samples' storage modulus (G') and loss modulus (G'') gradually increased with increasing sweep frequency. The G'' value was significantly lower than the G', indicating that the starch gels with or without QBG had good viscoelasticity. All the tested samples were typical weak gel systems (Chen et al., 2014).

The elastic modulus of the starch gel system containing QBG with different molecular weights and concentrations was lower than the pure RS gel system. This may be because QBG enhances the entanglement between β-glucan molecules and molecular starch chains in the gel system, increasing the network density of the gel system. As a result, the energy storage modulus [G'] and loss modulus [G''] were RS/LQBG > RS/MQBG > RS/HQBG. According to our previous study, QBG behaves in an aqueous solution as a pseudoplastic fluid with high viscosity, which may have caused a reduction in the elastic modulus and increased the viscosity of the gel system. However, the viscosity of all the RS/QBG samples was lower than RS alone. Possibly, the high viscosity of β-glucan inhibited the starch gelatinization. Furthermore, gelation is challenging because of the glucan chain's consecutive β-(1 → 4)-linkage blocks. This characteristic may influence the gelling capacity of the RS/QBG. Moreover, competition between QBG and water, then QBG wrapping around the starch granules may decrease RS's ability to gel, thus inhibiting the gelatinization and digestibility of rice starch. Amylose is the primary polymer used to create cross-linked networks. Therefore, we hypothesized that QBG was connected to leached amylose, which could slow the reaction between starch amylose molecules, subsequently delaying amylose re-aggregation and reducing the elasticity and viscosity of the starch paste.

### XRD

**Fig. 3:**
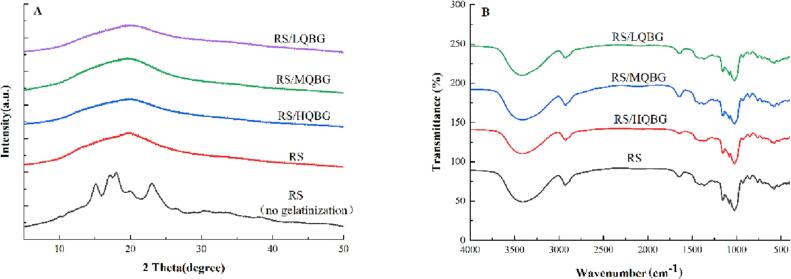
X-ray diffraction patterns (A) and fourier transform infrared spectra (B) of RS and RS/QBG with a β-glucan concentration of 5.0% RS. RS, rice starch; LQBG, low *Qingke* β-glucan; MQBG, medium *Qingke* β-glucan; HQBG, high *Qingke* β-glucan.

The XRD patterns of RS and RS/QBG gels are shown in Fig. 3A. The analysis revealed that gelatinized RS showed a typical A-type pattern, which typically had high-intensity peaks at 15°, 17°, 18°, and 23° and is common characteristic of RS (Hung et al., 2016).

The natural A-type RS pattern was lost during the pasting test, demonstrating that the RS in the pasta was entirely gelatinized. RS/QBG showed a weak absorption peak at near 20° and the diffraction was reduced and became wide compared with RS. This may be attributed to the inhibitory effect of *Qingke* β-glucan molecules on the binding of starch molecules, which consequently reduces the amylose content in the system and diminishes the interaction between starch molecules. The addition of β-glucan weakened the recrystallization of the starch molecular chains, consistent with the decrease in the gelatinization enthalpy (*ΔH*) of RS/QBG.

### FTIR

The interaction between β-glucan and starch was evaluated by FTIR spectroscopy. The infrared spectra of the RS/QBG samples are shown in Fig. 3B. No new absorption peaks were observed in the RS/QBG samples compared to the RS control group. This indicated that β-glucan did not bind to RS molecules but was bound through hydrogen bonding or hydrophobic interactions. Considering the structural characteristics of β-glucan and starch molecules, we speculated that β-glucan and RS molecules mainly interact through hydrogen bonding. QBG and RS are polysaccharides containing many hydroxyl groups; the principal absorption peaks were located near 3408 cm^−1^ (stretching vibration of the hydrogen bonding –OH groups) (He et al., 2020). The crystal zone of starch has an ordered structure, and its absorption peak is approximately 1047 cm^−1^. The absorption peak in the amorphous region is approximately 1022 cm^−1^. The ratio of the absorption peak intensity at 1047 cm^−1^ to 1022 cm^−1^, *R*_1047_/_1022_, can be used to characterize the short-range ordered structure of the sample, reflecting the degree of aging of starch. The larger the ratio, showed the higher the order of starch molecules, the greater the crystallization, and the more pronounced the regeneration. The *R*_995_/_1022_ value indicates changes in the double helix structure within the starch granules. A higher *R*_995_/_1022_ value corresponds to a more pronounced double helix structure.

Compared with the original RS, the *R*_1047_/_1022_ and *R*_995_/_1022_ values of RS/QBG decreased (Table 2), indicating that the addition of β-glucan could inhibit the formation of the short-range ordered structure of starch and delay aging. Furthermore, because the interaction between QBG and amylose was more substantial than that between amylose and amylose, QBG prevented the leaching of amylose from forming hydrogen bonds, delaying the short-term retrogradation of rice starch.

**Table 2:** Short-range ordered structures (*R*_1047/1022_ and *R*_995/1022_), gelatinization parameters and digestibility of RS and RS/QBG with β-glucan of different molecular weights and concentrations.

Samples	*R*_1047/1022_	*R*_995/1022_	*T*_o_ (°C)	*T*_p_ (°C)	*T*_c_ (°C)	Δ*H* (J/g)	RDS (%)	SDS (%)	RS (%)
RS	1.1138 ± 0.0051*^a^*	1.1743 ± 0.0023*^a^*	63.3 ± 0.3*^a^*	69.5 ± 0.3*^a^*	74.8 ± 0.3*^a^*	8.1 ± 0.1*^a^*	89.8 ± 0.8*^a^*	5.2 ± 0.1*^d^*	5.0 ± 0.1*^f^*
RS/2.5 %LQBG			63.3 ± 0.3*^a^*	69.4 ± 0.5*^a^*	74.6 ± 0.4*^a^*	7.9 ± 0.1*^ab^*	86.6 ± 0.9*^b^*	6.4 ± 0.1*^c^*	7.0 ± 0.2*^e^*
RS/2.5 %MQBG			63.4 ± 0.5*^a^*	69.2 ± 0.4*^a^*	74.7 ± 0.3*^a^*	7.8 ± 0.1*^ab^*	86.3 ± 0.7*^b^*	6.6 ± 0.2*^c^*	7.1 ± 0.2*^e^*
RS/2.5 %HQBG			63.6 ± 0.4*^a^*	69.6 ± 0.3*^a^*	75.3 ± 0.5*^a^*	7.7 ± 0.1*^b^*	85.5 ± 0.8*^b^*	6.7 ± 0.2*^bc^*	7.8 ± 0.2*^d^*
RS/5.0 %LQBG	1.0385 ± 0.0042*^b^*	1.0870 + 0.0032*^b^*	63.6 ± 0.3*^a^*	69.7 ± 0.4*^a^*	74.8 ± 0.3*^a^*	7.3 ± 0.2*^c^*	83.4 ± 0.7*^c^*	7.0 ± 0.1*^b^*	9.6 ± 0.2*^c^*
RS/5.0 %MQBG	1.0276 ± 0.0028*^c^*	1.0601 ± 0.0021*^c^*	63.5 ± 0.5*^a^*	69.4 ± 0.5*^a^*	75.3 ± 0.4*^a^*	7.1 ± 0.2 *^cd^*	81.2 ± 0.7*^d^*	7.5 ± 0.1*^a^*	11.3 ± 0.2*^b^*
RS/5.0 %HQBG	1.0153 ± 0.0032*^d^*	1.0539 ± 0.0043*^d^*	63.3 ± 0.4*^a^*	69.4 ± 0.4*^a^*	75.1 ± 0.5*^a^*	6.9 ± 0.1*^d^*	79.5 ± 0.8*^e^*	7.8 ± 0.2*^a^*	12.7 ± 0.2*^a^*

RS, rice starch; LQBG, low *Qingke* β-glucan; MQBG, medium *Qingke* β-glucan; HQBG, high *Qingke* β-glucan; *R*1047/1022, the ratio of absorbance 1047 cm^−1^/1022 cm^−1^ in FT-IR spectra, respectively; *R*995/1022, the ratio of absorbance 995 cm^−1^/1022 cm^−1^ in FT-IR spectra, respectively. *T*_o_, starting temperature; *T*_p_, peak temperature; *T*_c_, termination temperature; Δ*H*, gelatinization enthalpy; RDS, rapidly digestible starch; SDS, slowly digestible starch; RS, resistant starch. Results are presented as the mean ± SD. Values with different lowercase letters in the same column represent significant differences (p < 0.05).

### Thermal properties

Table 2 lists *T*_o_, *T*_p_, *T*_c_, and *ΔH* as computed from the DSC thermograms. QBG addition did not considerably affect the magnitudes of *T*_o_, *T*_p_, and *T*_c_ for RS and gelatinization of starch samples.

This result aligns with the finding that β-glucan has little impact on gelatinization temperature (Banchathanakij & Suphantharika, 2009). The energy-ordered structure of starch granules can be characterized by gelatinization enthalpy (*ΔH*) (Luo et al., 2020). According to DSC, the *ΔH* of the RS/QBG samples decreased compared to RS. Statistical analysis of DSC exhibited that *ΔH* of RS/5.0 %HQBG (6.9 J/g), RS/5.0 %MQG (7.1 J/g), RS/5.0 %LQBG (7.3 J/g), RS/2.5 %HQBG (7.7 J/g), RS/2.5 %MQBG (7.8 J/g) and RS/2.5 %LQBG (7.9 J/g) were lower than RS (8.1 J/g). The amount of heat required was reduced owing to the preservation of RS crystallization by β-glucan. The enthalpy (*ΔH*) order was RS/LQBG > RS/MQBG > RS/HQBG and RS/2.5 %QBG > RS/5.0 %QBG, suggesting that the tightness of starch granules wrapped by β-glucan could be increased by increasing the molecular weight and concentration of QBG. QBG may have promoted excellent bonding with the starch granules owing to its higher viscosity, especially at higher concentrations and molecular weights. This enhanced bonding likely improves QBG's ability to protect the starch crystallization zone. Moreover, the enhanced hydration capacity of QBG inhibits the movement of water molecules and prevents them from penetrating the starch granules (Krüger et al., 2003, Ma et al., 2019). Therefore, QBG has the potential to be an effective ingredient in food and other industries where high shelf life and stability are desirable.

### SEM and CLSM analysis

**Fig. 4:**
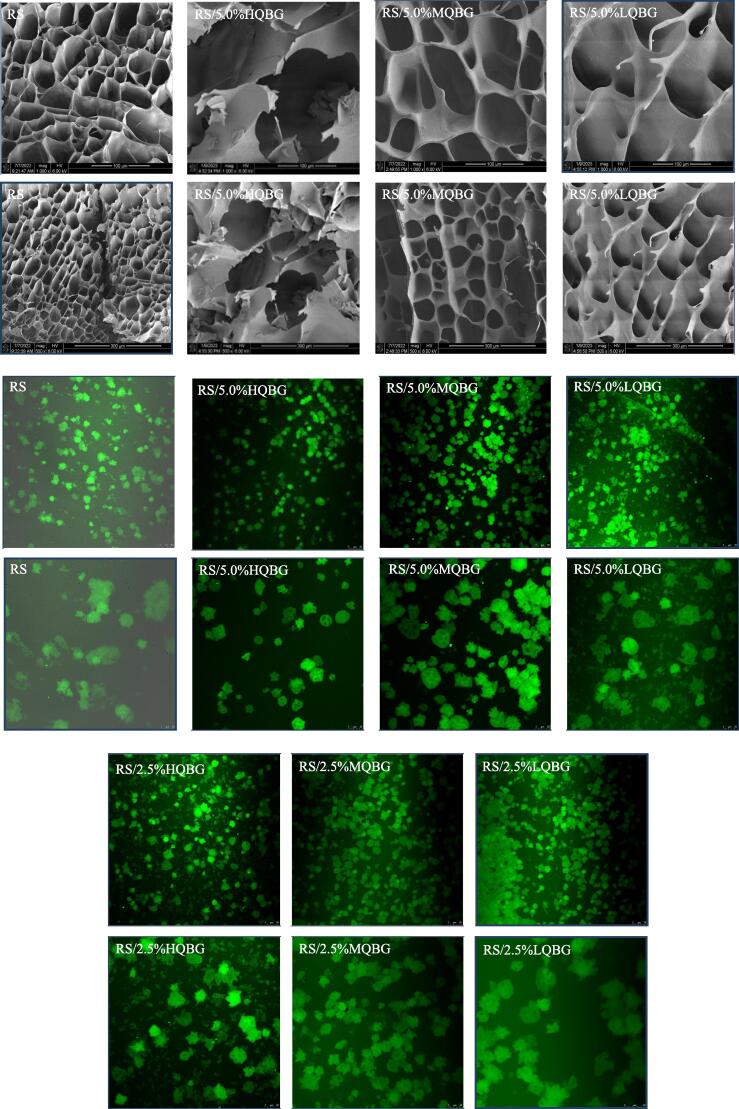
Scanning electron microscopy images and confocal laser scanning microscopy images of RS and RS/QBG with β-glucan of different molecular weights and concentrations. RS, rice starch; LQBG, low *Qingke* β-glucan; MQBG, medium *Qingke* β-glucan; HQBG, high *Qingke* β-glucan.

Fig. 4 shows micrographs of freeze-dried RS and RS/QBG gels at a concentration of 5.0%. Microscopic evaluation of the network structure of the RS/QBG gels was performed at magnifications of 1000X and 500X. The cellular architecture of all the samples resembled the β-glucan-pea starch gels (Cui et al., 2023), and the pore size and shape varied among the samples.

The RS samples exhibited thicker and denser pore walls and considerably smaller pore sizes than the RS/QBG samples. The internal structure of RS/QBG was loose, less dense, and more brittle, with more cracks and disordered fragments as the molecular weight of QBG increased. Compared with the RS/MQBG and RS/LQBG gels, the pores in the RS/HQBG gels were uneven and large. These results show that RS/QBG with larger molecular weights has better van der Waals forces. Therefore, larger slices were observed for the RS/HQBG gel than for the RS/MQBG and RS/LQBG gels. It is possible that QBG absorbed water and expanded before starch gelation, thereby competing with the starch for water. A large amount of QBG was wrapped in starch, which damaged the stability of the starch gel network. This outcome is consistent with the findings of a previous study in which the addition of hydrophilic colloids inhibited water absorption expansion and the exudation of amylose from RS granules (Banchathanakij & Suphantharika, 2009).

Fig. 4 shows confocal laser scanning microscopy (CLSM) images of the 5% RS gels with and without QBG. RS gels frequently contain remnants of starch granules or ghosts. A starch ghost is an envelope formed by starch granules after the leaching of starch components during heating with adequate water (Nagano et al., 2008). RS/QBG gels showed fewer starch ghosts than RS gel. Amylose leaching was limited, and the granule border was considerably cleaner in the RS/QBG systems than in the RS control. RS/5.0 %QBG had less leaching of starch components from starch granules and cleaner borders than RS/2.5 %QBG at the same molecular weight. RS/HQBG had less leaching of starch components from starch granules and cleaner borders than RS/MQBG and RS/LQBG at the same concentration. This inhibited starch gelatinization and reduced the peak viscosity of starch gelatinization, as indicated by the pasting properties and SEM results.

Previous studies have explained how starch molecules in grains are interconnected via hydrogen bonds (Nagano et al., 2008, Qiu et al., 2016). In the pure starch system, water molecules penetrate the starch particles during heating, resulting in different osmotic pressures, breakdown of hydrogen bonds, and release of internal starch polymeric groups. Studies have demonstrated that some hydrophilic colloids, such as guar gum, can cover the starch particle surface, acting as a protective agent or lubricating layer and protecting the starch particles (Nagano et al., 2008). When dissolved, amylose can interact with hydrophilic colloids to form a dense phase around swollen starch particles (Funami et al., 2008). QBG molecules protected the granule structure during gelatinization, similar to a previously reported hydrophilic colloid system. Macromolecular interactions may involve intermolecular hydrogen bonding and chain entanglements.

### *In vitro* digestion characteristics

Table 2 lists the rates of RDS, SDS, and RS formation during the digestion. As the concentration and molecular weight of QBG increased, RDS content decreased. In contrast, the SDS and RS contents exhibited a increase in the RS/QBG samples compared with the RS sample.

The inhibition of the RS starch digestion rate was in the order of control < 2.5 %QBG < 5.0 %QBG. QBG affects the overall viscosity of the system, and an increase in QBG concentration can increase the viscosity of the entire system, thereby preventing starch form absorbing water (H. J. Kim & White, 2013). Another study found that dietary fiber inhibits starch digestion by creating a fiber network and reducing the susceptibility to enzyme attack (Regand et al., 2011).

At a constant concentration, the order of starch digestion was as follows: LQBG > MQBG > HQBG. It was found that incorporating QBG of higher molecular weight in mixture significantly increased the viscosity. This increase in viscosity may have reduced the water available for starch gelatinization, ultimately decreasing the susceptibility of the enzymes to digestion (Zheng et al., 2021). Moreover, partially ungelatinized starch caused by QBG of higher molecular weight could contain some ordered starch molecules, thereby causing poor physical accessibility to digestive enzymes (Zheng et al., 2021).

In addition, the wrapping of RS granules by QBG made some starch granules difficult to digest during the pasting process. Moreover, QBG can compete with rice starch for water molecules, inhibiting the expansion of rice starch particles, and leading to incomplete gelatinization of some rice starch particles, thus reducing the digestion rate of rice starch that was observed in the pasting properties (Fig. 1), rheological analysis (Fig. 2), and CLSM (Fig. 4). Our results demonstrated that QBG addition had an inhibition effect on the digestion rate of rice starch, thereby assisting people with diabetes in controlling postprandial glucose levels in the future.

## Conclusions

This study demonstrated the effect of QBG with different molecular weights and concentrations on the pasting, rheological, structural, and digestion properties of RS/QBG gels. The peak, final, and setback viscosity values decreased when the β-glucan was added. This may be attributable to QBG wrapping around the starch granules, thereby preventing their gelatinization and expansion. According to rheological analysis, QBG reduced the dynamic modulus of the RS/QBG gels. The blended system's gelatinization enthalpy (*ΔH*) decreased with QBG addition. Compared with the original RS, the *R*_1047_/_1022_ values of RS/QBG reduced from 1.1138 to 1.0153 and *R*_995_/_1022_ values of RS/QBG reduced from 1.1743 to 1.0539, respectively, indicating that the addition of β-glucan could inhibit the formation of the short-range ordered structure of starch and delay aging. XRD analysis showed the peak intensity did not vary significantly after the increase in the molecular weight of QBG. SEM and CLSM analyses revealed that the leaching out of the starch components was inhibited by QBG in RS/QBG. In addition, *Qingke* β-glucan increased the SDS and RS contents, decreased the RDS content, and decreased the digestion rate of the RS/QBG mixture system. The content of slowly digestible starch and resistant starch of the RS/QBG with the 5.0% HQBG addition were 7.8% and 12.7% respectively, which were 1.5 times and 2.54 times of the RS, respectively. These results may provide a theoretical basis for developing functional foods based on a starch/non-starch polysaccharide blending system.

## CRediT authorship contribution statement

**Lan Zhao:** Methodology, Visualization, Investigation, Writing – original draft. **Xinyan Jin:** Investigation. **Jia Wu:** Writing – review & editing. **Huibin Chen:** Supervision, Conceptualization, Writing – review & editing.

## Declaration of Competing Interest

The authors declare the following financial interests/personal relationships which may be considered as potential competing interests: Huibin Chen reports financial support was provided by Fujian Normal University.

## Data Availability

Data will be made available on request.
